# Pulmonary Peak Flow Assessment: An Easy Tool for Cardiac Output Estimation in Hemodynamically Stable Neonates

**DOI:** 10.1155/ijpe/8395241

**Published:** 2026-01-08

**Authors:** Angela Alfarano, Roberto Marzollo, Serena Amighetti, Cesare Tomasi, Maria Ilaria Bosio, Salvatore Aversa, Elena Borelli, Mario Motta, Francesco Maria Risso

**Affiliations:** ^1^ Department of Neonatology and Neonatal Intensive Care, Children′s Hospital, ASST Spedali Civili of Brescia, Brescia, Italy

**Keywords:** echocardiography, neonatal intensive care unit, preterm infants, right ventricular output, systemic blood flow

## Abstract

**Objectives:**

Right ventricular output (RVO) assessment can estimate systemic blood flow (SBF) in neonates but requires advanced echocardiography expertise. Pulmonary peak flow (PPF) could be an easier surrogate for RVO. This study is aimed at evaluating the correlation between RVO and PPF in neonates and assessing their intraobserver variability.

**Methods:**

In this single‐center, longitudinal, observational study, we included term and preterm neonates admitted to our Neonatal Intensive Care Unit between February and July 2022. A neonatologist with experience in functional echocardiography recorded targeted neonatal scans. Recordings were analyzed off‐line for echocardiographic measurements. After 12 months, a second analysis of the recordings was performed by the same examiner to avoid recall bias. We compared the two analyses for intraobserver variability assessment.

**Results:**

We analyzed a cohort of 33 term and preterm infants. We found a strong positive correlation between RVO and PPF (*p* < 0.001). RVO showed a mean intra‐observer difference of 34.2 mL/kg/min; PPF showed a mean intraobserver difference of 2.2 cm/s.

**Conclusions:**

Our findings support the use of PPF as a surrogate for RVO for cardiac output estimation in term and preterm neonates. Because of its simplicity, rapidity, and low intraobserver variability, PPF could be a useful tool for SBF assessment. Future studies should be performed to evaluate the correlation between PPF and other echocardiographic markers of low SBF.

## 1. Introduction

Low cardiac output states have been linked to a variety of negative effects in neonates [1]. Neonatologist‐performed echocardiography has gained increasing attention in recent decades; several studies demonstrate that it can be used for cardiovascular compromise evaluation and for hemodynamic management of critically ill newborn infants, especially preterm infants [2, 3].

Right ventricular output (RVO) assessment is one of the available echocardiographic methods to estimate systemic blood flow (SBF) in neonates [2, 4]. RVO reflects blood returning from systemic circulation and is not affected by ductal shunt [3, 4]. RVO measurements are confounded by the presence of a large left‐to‐right interatrial shunt; however, typically atrial shunts are smaller than ductal shunts in neonates [4]. Therefore, in the absence of significant interatrial or interventricular shunting, RVO reflects the cumulative inflow of deoxygenated blood and hence venous return, providing an estimation of SBF in neonates [3, 4]. Like all echocardiographic measurements, RVO provides noninvasive real‐time results. Previous studies have demonstrated the feasibility and reliability of RVO assessment in term and preterm infants [5]. However, RVO assessment shows a wide interobserver and intraobserver variability, mainly due to pulmonary valve (PV) diameter measurement [6–8].

Pulmonary peak velocity flow (PPF) measurement, normally used to estimate PV stenosis, was suggested as a simple screening tool for low SBF state [9, 10]; unlike RVO, it shows a low inter‐observer variability [8–10]. Only a few studies analyzed the correlation between PPF and RVO. A relationship between low RVO (< 150 mL/kg/min) and PPF < 0.45 m/s was first described by Evans [9]. Another recent study showed the association between PPF < 0.4 m/s with reduced tricuspid annular plane systolic excursion (TAPSE) and lower RVO [11]. PPF showed a good prognostic accuracy in predicting high‐grade intraventricular hemorrhage in preterm infants, noninferior to RVO and superior vena cava flow, suggesting that this measurement could be used in clinical practice [10].

The purpose of our study is to describe the relationship between RVO and PPF in term and preterm infants admitted to the neonatal intensive care unit (NICU); we also evaluated RVO and PPF intraobserver variability.

## 2. Materials and Methods

This is a pilot, longitudinal, observational study conducted at a tertiary NICU over a 6‐month period (February to July 2022). The study was approved by the institutional research ethics boards. Newborn infants of any gestational and postnatal age and weight admitted to the NICU were included. We excluded newborn infants with congenital heart disease except for foramen ovale and patent ductus arteriosus (PDA). We also excluded patients with cardiovascular compromise requiring inotropes.

After parents or guardians informed consent, each enrolled neonate received a single targeted echocardiographic evaluation, which was digitally stored. Images rated poor (Score 4) or unusable (Score 5), according to Colan et al. [12], were excluded from the analysis. Five consecutive cardiac cycles were digitally stored for off‐line analysis. PV diameter and pulsed‐wave Doppler profile of pulmonary flow were assessed from a tilted parasternal long‐axis view, according to the institutional protocol. The internal PV diameter was measured at basal leaflets insertion, at end‐systole during off‐line analysis, and the average of three measurements was registered. VTI and PPF were manually traced and a mean of three measurements was registered [8]. In case of turbulent flow due to PDA at PV level, electrocardiogram trace was useful to better identify end‐systole. All echocardiograms were performed by expert neonatologists with more than 5 years′ experience in functional echocardiography.

The recorded images were analyzed by the same neonatologist at Time 0 and after 12 months to avoid recall bias. We described the correlation between RVO and PPF obtained in offline assessments and compared the measures at 0 and after 12 months to describe intraobserver variability.

### 2.1. Statistical Analyses

The database was formatted using Microsoft Excel Ver. 365 software and later imported into IBM SPSS software Ver. 27.1. Stata Ver. 17.0 software for comparisons or implementation of test outputs was also used.

Newborn infants′ demographic characteristics and ultrasound measurements were analyzed and reported using descriptive statistics. Continuous variables were subjected to the Kolmogorov–Smirnov test to assess Gaussian normality of sample distribution. Data were expressed as median (range) for nonparametric variables, mean and standard deviation (SD) for parametric variables, and percentage for categorical variables.

Student *t*‐test or analysis of variance (ANOVA) was used to compare the groups of continuous variables with normal distribution. Mann–Whitney test and Kruskal–Wallis test were used to compare the medians between groups of continuous variables with asymmetric distribution. Pearson′s correlation test and generalized linear models were used to assess associations between variables. Comparison and association analyses were considered statistically significant for *p* values < 0.05. This is a pilot study; therefore, no formal sample size calculation was done.

## 3. Results

From February to July 2022, we enrolled 35 newborn infants. We excluded two patients due to the presence of congenital heart defect (muscular ventricular septal defect). We evaluated recorded echocardiographic scans from 33 neonates; 18/33 neonates (54.4%) were born preterm (< 37 weeks). Seventeen neonates (51.5%) were scanned in the transitional period (first 72 h of life). Twelve patients (36.4%) had a PDA, but none of them required treatment at the time of echocardiographic scan. Detailed demographic data are described in Table 1.

**Table 1:** Clinical characteristics of the study population (33 neonates): mean and standard deviations (SDs).

**Gestational age, weeks, mean (±SD)**	**35.3 (± 4.86)**
Corrected gestational age at scan, weeks, mean ± (SD)	36.42 (± 4.64)
Birthweight, grams, mean (±SD)	2337.55 (± 1050)
Weight at scan, grams, mean (±SD)	2347 (± 1015)
Very low birth weight, number of neonates (%)	9 (27.2)
Spontaneous breathing, number of neonates (%)	20 (60,6)
Continuous positive airway pressure, number of neonates (%)	7 (21.2)
Non‐invasive Ventilation, number of neonates (%)	4 (12.1)
Invasive mechanical ventilation, number of neonates (%)	2 (6.1)
Male, number of neonates (%)	21 (63.6)

**Figure 1:**
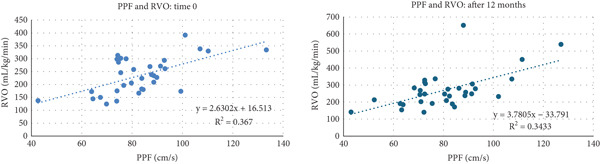
Interpolating graphs with coefficient of determination and regression equation in 33 neonates.

We found a strong correlation between PPF and RVO and between PPF and VTI, both in the first and second assessments performed 12 months later (Table 2 and Figure 1). Conversely, we did not find a correlation between PV and PPF (Table 2).

**Table 2:** Pearson′s correlation coefficient between PPF and RVO in 33 neonates.

**Variables**	**PPF**	
	**Pearson′s coefficient: Time 0**	**Pearson′s coefficient: after 12 months**
PV	−0.120	−0.086
VTI	0.885 ^∗^	0.904 ^∗^
RVO	0.606 ^∗^	0.586 ^∗^

Abbreviations: PPF, pulmonary peak flow velocity; PV, pulmonary valve; RVO, right ventricular output; VTI, velocity time integral.

^∗^ *p* < 0.001.

Analysis stratified by the presence or absence of PDA showed a similar correlation between PPF and RVO in the first and second assessments (Table 3).

**Table 3:** Pearson′s correlation coefficient between PPF and RVO measurements in open and closed PDA subgroups.

**Variables**	**PPF**			
	**OPEN PDA (12 neonates)**		**CLOSED PDA (21 neonates)**	
	**Pearson′s coefficient Time 0**	**Pearson′s coefficient after 12 months**	**Pearson′s coefficient Time 0**	**Pearson′s coefficient after 12 months**
RVO	0.652 ^∗^	0.693 ^∗^	0.538 ^∗^	0.511 ^∗^

Abbreviations: PDA, patent ductus arteriosus; PPF, pulmonary peak flow velocity; RVO, right ventricular output.

^∗^ *p* < 0.05.

The correlation between PPF and RVO was influenced by the day of life and was stronger after 72 h of life (Table 4).

**Table 4:** Pearson′s correlation coefficient between PPF and RVO measurements before and after 72 hours of life.

**Variables**	**PPF**			
	**< 72 h of life (17 neonates)**		**≥ 72 h of life (16 neonates)**	
	**Pearson′s coefficient Time 0**	**Pearson′s coefficient after 12 months**	**Pearson′s coefficient Time 0**	**Pearson′s coefficient after 12 months**
RVO	0,474	0,437	0,651 ^∗∗^	0,604 ^∗^

Abbreviations: PPF, pulmonary peak flow velocity; RVO, right ventricular output.

^∗^ *p* < 0.05 and  ^∗∗^ *p* < 0.01.

Table 5 shows intraobserver variability for PV, VTI, RVO, and PPF. All measurements showed a statistically significant difference, but the mean values are similar.

**Table 5:** Intraobserver variability for PV, VTI, RVO, and PPF in 33 neonates.

**Variables**	**Time 0 mean (±SD)**	**After 12 months mean (±SD)**	**Mean difference**	**p** **value**
PV (mm)	6.17 (± 1.53)	6.35 (± 1.42)	0.18	0.03
VTI (cm)	12.4 (± 2.84)	13.23 (± 3.21)	0.83	0.000
RVO (mL/kg/min)	235.6 (± 69.53)	269.8 (± 107.3)	34.2	0.004
PPF (cm/s)	82.5 (± 16.7)	80.3 (± 16.6)	2.2	0.001

Abbreviations: PPF, pulmonary peak flow velocity; PV, pulmonary valve; RVO, right ventricular output; VTI, pulmonary flow velocity time integral.

## 4. Discussion

This longitudinal observational study was designed to evaluate the relationship between PPF and RVO measurements in a cohort of 33 term and preterm infants admitted to our tertiary NICU.

RVO is a functional measurement of SBF state in the absence of anatomical heart defects; it could be used to monitor critically ill newborns because it provides a reliable estimation of cardiac output even in the presence of PDA, helping to address the choice of the right pharmacological therapy. However, RVO assessment shows a wide inter‐observer and intra‐observer variability. Unlike RVO, PPF is simple to obtain because it does not require measurement of PV diameter, which represents the largest source of error in RVO assessment [6–8].

Evans et al. [9] described a correlation between RVO < 150 mL/kg/min and PPF < 0.45 m/s in the first 48 h after birth; he found that a PPF of less than 0.35 m/s is strongly associated with an RVO of less than 120 mL/kg/min. Therefore, he suggested the use of PPF as a screening tool for low SBF status.

A correlation between PPF and RVO was also described by Joye et al. [11]. In this study, a cohort of 186 neonates with a median gestational age of 28.5 weeks was analyzed. A subgroup of 17 preterm infants, who had echocardiographic scans in the first 3 days of life, showed low PPF (< 0.4 m/s); a significant association between PPF < 0.4 m/s and lower TAPSE and RVO was described. Interestingly, these associations were found both in the presence and absence of a significant left‐to‐right shunting PDA, a common potential confounder in preterm neonates. The authors found that the pattern of low PPF typically occurred in acutely sick neonates, early in their postnatal course, suggesting that low velocity traces could alert clinicians to the underlying RV systolic dysfunction [11].

In this study, we found a strong correlation between PPF and RVO. In our cohort, no infants showed a PPF < 0.4 m/s and only one infant showed a PPF < 0.45 m/s, so we cannot evaluate its correlation with low RVO previously described. However, the strong correlation found in our study suggests a significant decrease in PPF with the reduction of RVO.

We also recorded the presence or absence of PDA for subgroup comparison, because a left‐to‐right shunting PDA may affect the relationship between echocardiographic parameters of hemodynamic status. In our analysis, we found a strong correlation between PPF and RVO even in the presence of PDA, in line with previous findings; to note, none of the patients were receiving treatment for hemodynamically significant PDA, so we are unable to speculate on the PPF and RVO correlation in this situation.

The correlation between PPF and RVO was influenced by postnatal age; we found a stronger correlation between PPF and RVO after 72 h of life (Table 2) than in the transitional period. The little sample size can explain this finding; however, elevated vascular pulmonary pressure in the first days of life could slow down pulmonary flow through the vessel, leading to lower PPF without significant influence on RVO.

We found an intraobserver mean difference in RVO of 34.2 mL/kg/min, similar to the previously described inter‐observer mean difference (22.1 mL/kg/min) [8]. Unexpectedly, we found a statistically significant intra‐observer variability for PPF, while interobserver variability was lower in our previous research. The little sample size, which is a limitation of our study, may be the cause of this unexpected outcome. However, the mean intra‐observer difference between PPF was only 2.2 cm/s which can be considered irrelevant in clinical practice.

All neonatologists experienced clinical impairment of critically ill newborns during echo scans, caused by procedure‐induced stress in a clinically unstable patient. RVO assessment requires advanced echocardiographic skills and needs vessel size measurement, which can be time‐consuming. RVO is calculated by multiplying stroke distance and heart rate by cross‐sectional area; since vessel diameter is squared in this equation, small measurement errors can lead to substantial variability in RVO values [6]. Several studies described the association between other echocardiographic measurements (such as superior vena cava flow, atrial strain, or Tissue Doppler) and low SBF‐related clinical situations [13–15]. All these measurements require specific training in neonatologist‐performed echocardiography. Considering that it requires only a single echocardiographic view, we propose that PPF may constitute a more time‐efficient screening modality than RVO, despite the current lack of supporting evidence in the literature. Moreover, PPF could potentially be performed even by nonexpert operators with limited echocardiographic training and may provide a valuable screening tool for detecting low SBF states in emergency settings or in the presence of a suboptimal echocardiographic window.

The main limitation of our study is the small sample size, although similar to other studies [6, 7, 9]. This is a pilot study, so no formal sample size calculation was done. Current data could be used as a starting point in evaluating RVO and PF measurements training efficacy. None of the enrolled patients required treatment for PDA; future studies could be performed in a population that also includes neonates with hemodynamically significant PDA.

Additional studies should be performed to test PPF measurement in a wide subset of patients to evaluate the correlation between PPF and other markers of low SBF, such as TAPSE, longitudinal strain, strain rates, and tissue Doppler.

In the present study, we included only hemodynamically stable neonates who did not require inotropic support and exhibited no clinical signs or symptoms of low SBF. Future investigations will be necessary to establish whether our findings remain applicable in contexts of clinical instability, shock, or in critically deteriorating neonates.

## 5. Conclusion

Our study represents an attempt to find an easy and repeatable tool for low SBF states screening in term and preterm infants admitted to the NICU, which is particularly useful in emergency situations.

We found a strong correlation between PPF and RVO, regardless of PDA presence, even within the first 72 h of life; therefore, we suggest PPF as the easiest and quickest surrogate for RVO assessment. Given its simplicity, PPF could serve as a practical tool for SBF assessment, even for nonexpert operators following appropriate training.

## Ethics Statement

This study was performed in line with the principles of the 1964 Helsinki declaration and its later amendments or comparable ethical standards. Approval was granted by our Ethical Committee of Brescia, ASST Spedali Civili (Approval Number EC 5154).

## Consent

The parents or guardians of babies enrolled gave their written informed consent.

## Conflicts of Interest

The authors declare no conflicts of interest.

## Author Contributions

**Angela Alfarano, Roberto Marzollo, and Serena Amighetti:** conceptualization, methodology, investigation, data curation, writing – original draft, writing – review and editing. **Cesare Tomasi:** methodology, formal analysis, writing – review and editing. **Maria Ilaria Bosio, Salvatore Aversa, Elena Borelli, and Mario Motta:** writing − original draft, writing – review and editing. **Francesco Maria Risso:** resources, supervision.

## Funding

No funding was received for this manuscript.

## Data Availability

The datasets used and/or analyzed during the current study are available from the corresponding author on reasonable request.
